# Symptomatic Pathology of the Mouth

**Published:** 1885-03

**Authors:** Elton R. Smilie

**Affiliations:** San Francisco, Cal.


ARTICLE II.

### SYMPTOMATIC PATHOLOGY OF THE MOUTH.

ELTON R. SMILIE, M. D., SAN FRANCISCO, CAL.

From the earliest transmitted records of the curative art, the mouth, has been, with its adjuncts, consulted for reflex evidences to determine in the mind of the remedial practitioner the nature and location of disease; as the rehearsal of patients' impressions would naturally attract his attention to its indications, and from experience the observing student must have appreciated the reactive value of its symptoms. The receptive mouth with all the elementary distinctions of taste for tissue co-operative representation, would not be perfect for organic reciprocation in the expression of desire in the selection of food and gustatory satisfaction in digestive disposal, without a special reactive communication through which to signal ill effects. The possession of this power, which observation had established as a reality, was gradually developed into more extended knowledge of individual organic demonstration, when anatomy opened to view physiological effects from the combination in action of systematic parts. Over these functional reciprocations of want, supply, and effect, necessary for healthy sustenance, the will—or with cultivation, the mind-as commissary held control. But as the attractions of association became disseminated, drawing together individuals subject to varied climate and engendered habit influences, their adverse adoption, without consideration of cause and effect, led naturally to the multiplication of disease and confusion in the blending of mouth indications characteristic of organic impression and complications. In primitive simplicity the animal desires held ruling sway, and by appetite and passion, wrought through the prima via and genito urinal glandular connection, direct symptoms in reaction; but now with the multiplied complications of disease; there are in sequence interruptions and accessions in transmission that render a reliable systematic diagnosis difficult. But the inordinate use of the procreative organs has produced a retrograde action, so marked, that venereal and cancerous diseases, are, by metastasis often transferred to the mouth, as if in direct reproff for exampled correction of deleterious habits. Although, there are variations in the virulence and translated seat of attack, the inner periosteum of the mucous membrane attachment is usually the seat, and if there is a defective tooth its alveolar process becomes the central point of invasion, and by necrosed gangrene the sockets are quickly destroyed and its roots dislodged.

But, if in anticipation, the characteristics of the disease have been diagnosed correctly, with the first appearance of the fungoid and liver-hued protrusion of the gum attachment, the tooth should be extracted and sloughing caustics applied to the depths of its sockets and to the adjoining parts beyond the evidences of diseased action. If the osteo-sarcomatous cancer by translation, once gains full headway in its attack upon the superior maxillary bones it is hard to arrest, and as it bygangrenous action disintegrates the bony structure it is generally slower in progress than the venereal disease which by destroying the cartilaginous union of the bones, detaches them without injuring the integrity of their structure. That the diseases had a common origin is sufficiently clear from similar peculiarities developed in progress, the cause of deviation being rationally accounted for from hereditary and contingent impressions.

There are families with ancestral pedigrees which have been noted for simplicity in dietetic and other habits that insure freedom from the malignant fungoid diathesis, and we can reasonably suppose that there was a period in the offshoot colonization of races, in which self-supporting labor insured immunity from the taint; or if incurred before separation, the natural means for rehabitation from the excesses which have always followed as a sequence of over-populous association. From the colonistic regenerations it is difficult to detect the exact date which was noted for the inception of a new disease and the immediate causes that contributed to its development. Even at the present time, with the multiplicity of means and trained minds for tracing cause in contributing degrees to effect, contagious and epidemic diseases appear and for periods more or less protracted hold destructive sway, and although a large quanity of ink is expended in conjectural investigation, their periodicity is not deterred or destructiveness mitigated; and future generations will be as little benefited by the sage conclusions of the writers, as we are by those who discoursed upon the origin and cause of the Egyptian plague an advent of cancerous degeneration with its remote source from or alliance with venereal contagion. Yet, the criticial reading of all that is judiciously written is an incentive to thorough comparison, and serves by training the mind for analytical investigation,, to prepare it for a legitimate course of research.

Of four hundred and seventeen tumors and deep seated excrescences suppurated out with escharotics, nine were of osteosarcomatous type, with the periosteum of the jaws, palate and other adjunct bones implicated as the attractive cause of secondary translation from irritation of the gravid uterus promoted from indigestible substances taken into the stomach to gratify the cravings of a morbid appetite. With an ordinary endowment of reason and fear of pollution, one would naturally suppose that in anticipation of venereal and cancerous inflictions, the sensuous appetite would be held under restraint, for they are by far, the most disgusting and loathsome of human maladies; as the victims are forced to realize, living, the horrors of the tombs, post mortem decomposition, and endure, not only the involuntary aversion of their associates of the human species, but of the lower orders of domestic animals, and above all their own existence. Notwithstanding physicians had noted that the aching of defective and sound teeth was caused by metastatic translation during the period of utero-gestation, from some inherent source of irritation peculiar to the habits or condition of the individual affected, they gave no more heed for the detection of source or in search of the tissue of transfer than the sectarian religious devotee does to the rationale of viaticum ceremonies as passports of reality to places of future soul existence, rated theologically as intensely happy or miserable. Over-feeding and rich diet, were known to produce gout and a deposit of earthy salts in the joints of the ankles and toes, in connection with renal and urinal formations, in kind, agglutinated or phosphated together to obstruct the ducts and vasa valves of excretion, by the earliest medical writers. Yet, to the present day the drain passages by the feet, whether deep seated lymphatic ducts or glandular tissue of syphon gravitation operated upon by peristaltic ejective contraction with spincter regulators, remains a question of doubt. Although unrecognized, still, that there is an important drain existing for conducting effete matter through or from the excretions of the lower extremities terminating for discharge from the plantar surface and between the toes, the merest tyro in medical science must acknowledge with the exercise of his powers of experimental observation. That it is separate from the external or superficial lymphatic system is evident from the peculiar reactive effects produced when the septic matter is retained from their closure; local pains, and disease with varicose veins indicating the passive resistance of gravitation or valvular hindrance to active re-absorption for the implication of the vital organs. The anatomical funnel-form taper of the leg from the thigh to the knee, and calf to the ankle, shows a constructive design in furtherance of the drainage system; and at the same time presents the natural probabilities of a provision to guard against reflux action; and the diseases, and their position and sequent stages of development in foot and ankle with their slow upward progress, confirms to a degree of certainty not only the existence of valves but the power neccessary for their operation. The movements of the leg in locomotion from muscular expansion and contraction, with their peculiar leverage attachments, naturally pre-suppose the ejective requirements of compression in the alternations of extension and relaxation, which from circumscribed pecularities of the dependent limbs would react upon the tubular vessels for the conveyance of fluids, that did not possess the ringed protection of the arteries, which of themselves derive their systolic action from the heart. These combined actions imply for healthy support a certain degree of voluntary motion, and when deprived of it, from torpidy superinduced by stomach engorgement of over distention, or by indigestible food, as an inevitable consequence stagnation ensues, and in the unrelieved vessels effete deposits more or less pernicious and difficult to be removed by the resumption of healthy diet and exercise. If the contra-indications are persisted into an accumulative extent, that precludes relief from the power of physicial reaction promoted by the resumption of healthy requirements, by slow degrees local septic impression forces the natural barriers and from the accretion of disorganization reaches within the scope subject to more direct vital absorption. At this stage the glands of the mouth and throat with the mucous membrane show reactive signs of participation symtomatic of the nature of the disease. Although previously sufficiently marked for the detection by the experienced eye, the pathognomonic symptoms in revulsion give plain expression to the nature of the disease and organs affected in transition. The evidences of these forced retrograde operations of absorptive transfusion, are upon studied investigation easily traced, and established in the mind of nature's student as matters of fact, readily demonstrated to the senses. The wonder is, that even in negligence of omission, the adjuncts in the drainage of the feet should have remained so long unregarded in their importance upon the health of the general system as to be left to the thoughtless cleavage clippings of the "corn doctor."

In verification of the existence and fact importance of the sewerage drainage of the lower extremities vs. theory, we have only to consult the habits and dietetics peculiar to them, and in co-existant relation their symptomatic and transferred effects upon other organs and tissues. The podagra aberrans will illustrate in the flight of pain the parts implicated, which from characteristic effects directly indicate the cause of disease, and means to be adopted for the restoration of healthy action; and though the symptomatic wanderings can be traced the organs, ducts, and tissues surcharged in retention of matter to distentious inertia, which suspends the power of reciprocation in functional duty, and by over-taxing a class with vicarious action weakens the sequent correspondence necessary for the development and retention of health from equalized labor. The phlegmasia dolens of the ankle and lower leg, usually affecting women of middle and old age, with passive habits accustomed to a standing position with limited routine movements affording insufficient limb and general exercise for effective drainage, is another form of disease which becomes transferable from septic absorption. The primary characteristic symptoms indicate clearly the cause; and in the stage progress of the disease, from turgescence to dropsical ulceration, and its pari passu or sequent symptomatic varicose veins, can be traced the retention and presence of vitiated matter, that becomes for varying periods local, from its disorganizing effect upon the radiating vessels for venous distribution, which renders them powerless, in an increasing degree, to overcome the pressure of gravitation, that opposes the systole reaction of the heart and arteries upon the veins for the return current to its source of re-oxygenation. All of the diseases of the extremities in their acute inception, and until chronicism determines the nature, seat, and extent of the parts involved, reflect back to the mouth only symptomatic impressions without distinguishing features to determine more than that blood deterioration from resulting absorption of veins, introduced or generated from the retention of morbid matter, is the source of irritation. But, when in reaction the septic position reaches in accumulative process a vital organ, as the heart or lungs, with sufficient force to inoculate their tissues beyond the power of self-recuperation, in their special vocation of re-oxygenation, the valvular system of the fauces becomes affected from defective reaction of the blood, from the former's impaired action; and if the pulmonary system is the direct seat of the decomposing taint, the passages to and from the mouth become the drains of excretion. Even, the brain so essential to all the voluntary emotions of will dictation,—in secondary degree a dependant vital organ,—conveys its physical impressions of condition in confused symptomatic reflection through the avenues of the senses, to the adjuncts of the mouth, rather than by direct tissue influence; and can sustain for varying periods, hardening, softening and actual suppuration of one or more of its constituent parts without making its stage degrees of diseased progress distinctively manifest. But, although there are undoubtedly distinct evidences of reaction upon the mouth tissues, giving special indication of location and variations of disease, they have become so complicated from hereditary transmissions and malformations, that the impressions of organic and functional disturbances are so faintly shadowed that conjecture must enter largely into a diagnosis predicated entirely upon their symptomatic mouth demonstrations. In gout, when the gravitation drain has become obstructed to the knee, involving the passages and tissues of the joint, causing the absoption of the bursa mucosæ, oppression of the heart's action speedily follows from engorgement and loss of ventricular contractile power, the first effects in the series which foreshadow sudden death.

At this stage, the derangement of the valvular condition of the fauces becomes in a marked degree conspicuous, showing the intimate relation of their dependency, for integrity and promptness of action, upon the tonicity of the lungs and heart for the necessary elaboration and distribution of the blood. Phlegmasia dolens, in contradistinction to gout, has not an hereditary claim in transmission to either abundance or richness of diet as a latent cause of pre-disposition, but is rather an heir-loom inheritance of poverty and impoverished blood with entailed labor beyond the healthy powers of endurance.

The former disease having its origin in the generation of a specific obstruction, in generality begot of torpidity from engorgement or over-distension of the digestive, chyliferous and blood distributing vessels, and in cases of aggravation to peculiarities in food constituents or method of preparation. As in war, the rank and file of poverty are the common food of epidemics and diseases, self-generated from insufficient nutriment and laborious habits of exposure; while the rich in their exemption from necessity, create for themselves a class of obstructive diseases, that makes a bare competence gained with prudent discretion the reasonable allotment for the majority in life as a guard against the temptation to indulgence; but unfortunately the poor consider labor the curse of their condition and cultivate habits which render them slaves to their passions, and subject tools—with the snarling protest of self-imposed imbecility—to the controlling power held by the possessors of riches.

With these leading observations upon diseases subject to actual transfer, and those which are in fact only translated through septic taint forced to react upon the system from the obstruction of natural drains, which in free functional duty have hitherto passed unnoticed and unrevealed, other than by the general term of lymphatic vessels, it will be apparent that the mouth as the speaking centre of the adjunct senses, and symptomatic indicator of the entire organization, enacts directly and indirectly a very important part in fulfilling the conservative duties of commissary guardian, under brain control, for the selection and preparation of nutriment best adapted for the support of the animal economy in a healthy condition. It will also be equally clear to the educated perception, that the teeth of themselves, hold the same relation to the individual possessor that the millstones of the mill perform for the community at large; and can be substituted with artifical without serious detriment to the organic functions of the body. This, however, may be accounted an additional proof,—in negative operation—of man's superiority over the lower orders of animal life, who have neither the perfection of mouth adaptibility, *or necessity*, when free from human control, for artificial dentures; while at best the dentist only performs the service of a millright, and is no more worthy of a title for professional distinction, unless he makes the relations of the mouth with the rest of the system his study, and is able to take rational cognizance of its symtomatic phases of disease both direct and in reversion.

The method adopted by Miss———, a practitioner of dentistry in a prominent Russian city, for the eradication of an osteo-sarcomic tumor, and treatment of the diseased bone, will illustrate the importance of a full understanding of the anatomical, physiological, and pathological relations that exist between the mouth and the rest of the system. The essentials of her relation will attest to her natural and acquired ability, as well as to the delicacy and refinement of her disposition while enacting her part in a profession repugnant to the inborn instincts of woman; the adoption of her narrative style exultant in success, should not detract from its interest. Although addressed in personal confidence to the writer, its publication, from the important bearing its exhibits in extension from the simple mechanical treatment of the teeth will serve as a plea for his exculpation from a course which might otherwise be rated as a breach of trust.

——* * *——, Io triumphe! Permit me, my dear——, the privilege of using this excellent expression of joy on mutual account for a success which I hope you will think merited from the course I adopted to attain it; and pardon narrative prolixity, for in terse medical phrase I could not do justice to, or express my admiration for the rare qualities of mind possessed by my patient. But alas! that misled and misused she should feel anxious that the bitter experience of her life shall prove a warning to others; and it is with her permission and expressed wish, that with opportunity, I should make known and in no way extenuate the causes of her disease; for the distinction she has gained by her talents, wit and generosity, will prove more attractive for acquirement than the fear of her penalties will deter from a like course. Even with her proviso request, that I would only use her wayward name known to the public, curiosity will, if disposed, soon ferret out her real history and blazon it to the world with the insinuation of motives foreign to the natural instincts of her nature. Then I urged that silence with regard to name was the better policy of the medical attendant, she acknowledged a piquant curiosity to learn the very result I judged would prove so repugnant to the sensitive nature of a modest woman. But, probably, noting the shadow of disappointment that I could not repress from the expression of my face, she urged that otherwise the intelligent, whose good opinion she fondly cherished, might think she still lacked the discretion of refined delicacy that would be the natural proof that she possessed innately the qualities of judgment necessary to make the punishment she had received for the transgression of nature's laws useful, from sincerity of intention. To proceed in train; on the 5th of January '81, I received, at my operating rooms, a note from a lady of high rank, well-known to me from her reputation of being the most courtly and wittiest woman of the day in Europe, desiring a professional appointment for the evening. I was about to answer that gaslight was not suited for dental operations when it occurred to me that she was undoubtedly well acquainted with all the usages and mechanical requirements of the art, and probably had some other motive in view for the engagement. With this timely thought I named an hour most likely to suit her habits, and she was announced prompt to the minute, and for the unfashionable punctuality apologized by saying that it was caused by the selfish anxiety of fear; but instead of stating its source, and the nature of service required, with seeming Yankee avidity, as if anxious to adapt herself to the national peculiarities of my nativity, she gradually led the conversation, with an inquiring disposition into the bias transplanted from ancestral impressions, and when I stated that my mother's parents were natives of Ireland and my father's of New England she gave me, with a look of surprise, a keen scan of scrutiny. When she had taken an impression of my external miscegenic bearings she drew from me a relation of the habits of my childhood and the method adopted for my education. It was with a feeling tinge of self-deprecia- tion that I acknowledged that my preliminary education was derived solely from parental tuition, and medical and dental from your instruction yet I could perceive that it rather strengthened than abated the respectful warmth of her attention, and after commendation of my judgment, which she said denoted from its clearness the encouragement of thought from single leading instructive suggestions, instead of the diverse theoretical teachings of the schools. You may be sure, that with all my unworthiness, and the thought that her judgment must be partial, even with the interested motives that prompted her to test my natural and acquired ability, I felt flattered with the evident favorable impression I was making, which the cynical wag of your nose will not altogether abate.

*To be Continued.*

