# An Omission

**Published:** 1885-04

**Authors:** 


AN OMISSION.—In the preceding number of this journal, the portion of the article entitled "Symptomatic Pathology of the Mouth," through a mistake on the part of the printer, was not credited to the *Dental Practitioner* in which journal it originally appeared. We take pleasure in making this correction.

